# Polysubstance-Induced Vasoplegia Managed with Venoarterial Extracorporeal Membrane Oxygenation: A Case Report

**DOI:** 10.5811/cpcem.62370

**Published:** 2026-09-26

**Authors:** William J. Bleifuss, Aaron E. Robinson, Beth M. Heather, Hannah M. Brown, Tyler G. Floden, Jon B. Cole

**Affiliations:** * Hennepin County Medical Center, Minnesota Regional Poison Center, Minneapolis, Minnesota; † Hennepin County Medical Center, Department of Emergency Medicine, Minneapolis, Minnesota; ‡ Hennepin County Medical Center, Critical Care Nursing and the Extracorporeal Life Support Program, Minneapolis, Minnesota; § Hennepin County Medical Center, Department of Laboratory Medicine and Pathology, Minneapolis, Minnesota; ¶ Hennepin County Medical Center, Department of Critical Care Medicine, Minneapolis, Minnesota

**Keywords:** levomilnacipran, angiotensin II: vasoplegia, ECMO, case report

## Abstract

**Introduction:**

While the use of venoarterial extracorporeal membrane oxygenation (VA-ECMO) becomes increasingly commonplace in overdose, questions about its role in various shock states remain. The application of VA-ECMO with impaired myocardial function is well established, and use in cardiogenic shock precipitated by poisoning has continued to increase. In poison-induced vasoplegia with preserved myocardial function, the role of VA-ECMO remains undefined. Many atypical antipsychotic and antidepressant agents are known to cause mild hypotension via alpha-1 adrenergic blockade. Intravenous fluids and vasopressors alone are generally effective for single-substance ingestions. In the setting of polysubstance intoxication, synergism leading to bradycardia and refractory vasoplegia refractory to conventional therapies may result, prompting consideration for extracorporeal support.

**Case Report:**

A 71-year-old woman presented to the emergency department (ED) after being found obtunded near bottles of quetiapine, olanzapine, trazodone, mirtazapine, levomilnacipran, eszopiclone, and clonazepam. She underwent prehospital intubation and received push-dose epinephrine for hypotension. In the ED, the patient remained hypotensive with a relative bradycardia, requiring escalating doses of norepinephrine, vasopressin, epinephrine, angiotensin II, and methylene blue infusions. Her extremities remained warm, and point-of-care echocardiography revealed hyperdynamic function, consistent with vasoplegic shock. She subsequently underwent VA-ECMO cannulation, which was followed by a rapid decrease in vasopressor requirements and decannulation on hospital day 3. The patient recovered and was transferred to inpatient psychiatry neurologically intact. Urine mass-spectrometry revealed large peaks for quetiapine, olanzapine, milnacipran, mirtazapine, trazodone, and zopiclone.

**Conclusion:**

While overdose data are limited, levomilnacipran may precipitate or potentiate relative bradycardia with hypotension and a preserved ejection fraction. Significant ingestions and synergism with other agents causing peripheral alpha-1 antagonism may lead to hemodynamic instability refractory to conventional therapies. Extracorporeal life support may aid in recovery.

## INTRODUCTION

Venoarterial extracorporeal membrane oxygenation (VA-ECMO) is a rapidly deployable modality for hemodynamic support in the setting of cardiogenic shock, although utility in predominantly vasoplegic states remains less well established.^1^ Venoarterial extracorporeal membrane oxygenation is a well-described treatment for poisoning, with rates of cannulation consistently increasing in the United States.^2^ While cardiogenic shock from poisoning is a relatively common indication, the effectiveness of VA-ECMO in vasoplegia from overdose is poorly defined.^1^,^2^ Multiple antipsychotics and antidepressants have been shown to cause peripheral vasodilation via alpha-1 adrenergic antagonism, which may lead to hypotension.^3^ Vasopressors and crystalloid are generally effective, while mechanical circulatory support is rarely required.^3^,^4^

We present a case using VA-ECMO in the setting of refractory vasoplegia from quetiapine, olanzapine, trazodone, mirtazapine and, more novelly, levomilnacipran, a serotonin–norepinephrine reuptake inhibitor (SNRI) for which overdose data remain limited.

## CASE REPORT

An otherwise healthy 71-year-old woman with major depressive disorder and hypertension presented to the emergency department (ED) via paramedics after being found unresponsive by her husband. Empty bottles of quetiapine, olanzapine, trazodone, mirtazapine, levomilnacipran, eszopiclone, and clonazepam were nearby. Amlodipine-valsartan, propranolol and venlafaxine were also noted on her medication list. Initial prehospital vitals were as follows: blood pressure, 89/55 mm Hg; and heart rate, 72 beats per minute. She required bag-valve-mask ventilation due to severe bradypnea and hypoxemia with a peripheral capillary oxygen saturation of 80%. The patient was intubated and received a total of 400 mcg of push-dose epinephrine for hypotension. Vitals on ED arrival were as follows: blood pressure, 71/46 mm Hg; heart rate, 66 beats per minute; temperature, 33.9. ºC; and mechanically ventilated at 14 breaths per minute. A venous blood gas returned with a pH of 7.33 (reference range, 7.31–7.41)’ partial pressure of carbon dioxide, 44 mm Hg (41–51 mm Hg); and lactate, 1.0 mmol/L (0.7–2.1 mmol/L), later peaking at 7.3 mmol/L.

Electrocardiogram showed a normal sinus rhythm with a PR, QRS and QTc of 154, 96 and 572 milliseconds, respectively. Her extremities were warm with normal capillary refill, and point-of-care echocardiography revealed hyperdynamic function without tachycardia. Over the following 8 hours, vasopressor requirements escalated to include norepinephrine (0.7 mcg/kg/min), vasopressin (0.08 u/min), and later epinephrine (0.3 mcg/kg/min), and angiotensin II (80 ng/kg/min). With ongoing uptitration of vasoactive agents, a 2 mg/kg bolus of methylene blue followed by a 0.5 mg/kg/hr infusion was also provided with minimal improvement. Due to a national shortage, hydroxocobalamin was not available.^5^ High-dose insulin was ultimately deferred given her hyperdynamic cardiac function. Hydromorphone and midazolam infusions were administered for sedation. The medical intensive care unit and regional poison center were contacted to discuss the potential utility of extracorporeal support. Given concerns for further hemodynamic deterioration with ongoing escalation of vasopressor needs for refractory distributive shock, the patient subsequently underwent cannulation for VA-ECMO (Table).^6^

*CPC-EM Capsule*

What do we already know about this clinical entity?

*Atypical antidepressants and antipsychotics may cause hypotension via alpha-1 adrenergic blockade. Effects are usually mild and respond to fluids and vasopressors.*

What makes this presentation of disease reportable?

*Levomilnacipran has limited data in overdose. Negative inotropy and chronotropy have been reported in animal models, but effects in humans remain poorly described.*

What is the major learning point?

*Levomilnacipran may blunt the compensatory cardiac output in polysubstance-induced distributive shock. VA-ECMO may be a viable therapy for refractory vasoplegia.*

How might this improve emergency medicine practice?

*Recognition that levomilnacipran toxicity may cause bradycardia with or without a normal ejection fraction, and require significant hemodynamic support in overdose.*

There was expected color change of the circuit, with bright red blood on the return limb and dark blood on the drainage limb. Within minutes, blood in both limbs changed to a dark color. Membrane lung function and cannula positioning were reassessed and appropriate. Colors in the circuit ultimately returned to normal following discontinuation of methylene blue. Transthoracic echocardiogram revealed an ejection fraction of 70% with hyperdynamic function. Hemodynamics continued to improve, and vasopressor needs decreased to norepinephrine and vasopressin by the end of hospital day 1. She was decannulated on hospital day 3, extubated on day 5, and transferred to inpatient psychiatry on day 10 neurologically intact. Urine mass-spectrometry revealed large peaks for quetiapine, olanzapine and milnacipran, additionally noting the presence of ketamine (iatrogenic), mirtazapine, trazodone and zopiclone (Image).

## DISCUSSION

As VA-ECMO becomes increasingly available, applications in drug-induced circulatory collapse may continue to expand.^1^,^2^ Utility in vasoplegia remains unclear, with some data suggesting poor survival in comparison to cardiogenic shock.^1^ The decision to cannulate for VA-ECMO in these cases remains nuanced. The medications ingested, clinical course, supportive therapies, and patient comorbidities should all be taken into consideration. Consultation with a medical toxicologist, regional poison center, and intensivist or institutional shock team should be used. In more austere community settings, signs of early hemodynamic deterioration signaled by escalating vasopressor needs and clinical evidence of malperfusion should prompt consideration for transfer to a facility with advanced supportive therapies.

Several antipsychotics and atypical antidepressants may cause hypotension via blockade of peripheral alpha-1 adrenergic receptors, although single-substance overdoses are generally well tolerated and rarely require vasopressor support.^3^,^4^ Trazodone and mirtazapine are considered to be generally benign, potentially causing orthostasis, although trazodone has been associated with bradycardia and atrioventricular conduction delay.^7^,^8^ Quetiapine and olanzapine overdoses may cause clinically significant hypotension, the former more so than the latter.^3^,^9^ One case of isolated quetiapine poisoning required VA-ECMO, although this was driven by abnormal myocardial function and ventricular asynchrony.^10^ Racemic milnacipran and isomeric levomilnacipran toxicity remains poorly described. A single-substance milnacipran ingestion caused autonomic instability requiring an intermittent norepinephrine infusion, suspected serotonin syndrome, and stress-induced cardiomyopathy.^11^ In our patient, the area under the curve for mass spectrometry of quetiapine, olanzapine, trazodone, mirtazapine and milnacipran suggests possibly supratherapeutic concentrations. Notably, the largest peak corresponds to milnacipran, suggesting a significant serum concentration, although this was unable to be specifically quantified.

Our instruments are unable to distinguish between the dextro and levo isomers of milnacipran, hence the reported detection of milnacipran, not specifically the levomilnacipran the patient was prescribed. This same principle applies to the detected zopiclone in the setting of eszopiclone ingestion. While isolated alpha-blockade from any single agent may be effectively managed with vasopressors alone, given refractory hypotension, hyperdynamic myocardial function, and an exam suggestive of vasoplegia, synergism between multiple agents was possible. Trazodone may have contributed to the concurrent bradycardia. Bradycardia is less commonly documented in isolated olanzapine or quetiapine ingestions, as both peripheral alpha-1 blockade and antimuscarinic effects generally potentiate tachycardia. The specific effects of levomilnacipran remain unclear. The patient exhibited no clear signs of autonomic instability or serotonergic excess, although this may have been blunted by the administered sedative infusions to a certain degree. A previous report describes initial bradycardia and hypotension, similar to the case of our patient, although with a depressed ejection fraction (30–35%) and subsequent hypertension and tachycardia, which did not develop in our case.^11^

A canine model suggests large doses (10 mg/kg) may precipitate negative inotropic, chronotropic, and dromotropic effects, which may have prevented a reflexive tachycardia in the setting of distributive shock.^12^ While similar agents such as venlafaxine are known to cause cardiogenic shock, given evidence of hypercontractile myocardial function and warm peripheral extremities, our patient’s presentation was clinically consistent with primarily vasoplegia.^13^ Cases with a normal or depressed left ventricular ejection fraction, when combined with signs of peripheral vasodilation, are more suggestive of concomitant cardiogenic shock, particularly when a poison may be blunting compensatory tachycardia. While both trazodone and levomilnacipran may have contributed to concurrent bradycardia, the significantly larger peak of the latter on mass spectrometry suggests levomilnacipran may have played a more predominant role, although this remains inferential in the absence of serum concentrations.

The atypical color change noted in both limbs during cannulation was likely precipitated by the administered methylene blue. Its action as a dye with light-absorption along the orange-red spectrum may directly darken the color of blood.^14^ Methylene blue’s oxidant effects are also known to precipitate methemoglobinemia, although more commonly at doses approaching 7 mg/kg.^15^ While such color discrepancies should otherwise be interpreted as a potential sign of oxygenator failure, similar phenomena have previously been documented following methylene blue administration during VA-ECMO use.^14^ The monoamine oxidase inhibition exhibited by methylene blue may also warrant concern for precipitation of serotonergic excess or serotonin syndrome with concomitant selective serotonin reuptake inhibitor or serotonin and norepinephrine reuptake inhibitor use.^16^ Signs of excessive serotonergic activity such as worsening tachycardia, encephalopathy or agitation, and lower extremity hyperreflexia and clonus should be monitored, although in the critically ill patient, the more deleterious effects of musculoskeletal rigidity and subsequent hyperthermia may be blunted by sedatives, such as the midazolam infusion in our patient. No signs of serotonergic excess were evident on exam.

## CONCLUSION

While clinical experience in overdose is limited, levomilnacipran may precipitate or exacerbate hypotension and bradycardia without a depressed ejection fraction, potentiating malperfusion and shock, particularly when taken in conjunction with other vasoactive medications. Although VA-ECMO is not classically considered as a standard treatment for drug-induced distributive shock in the absence of myocardial dysfunction, it may be considered as a therapeutic option in cases refractory to vasopressor support or other salvage therapies,. Elucidation of definitive benefit remains an area requiring ongoing study.

## Figures and Tables

**Image:**
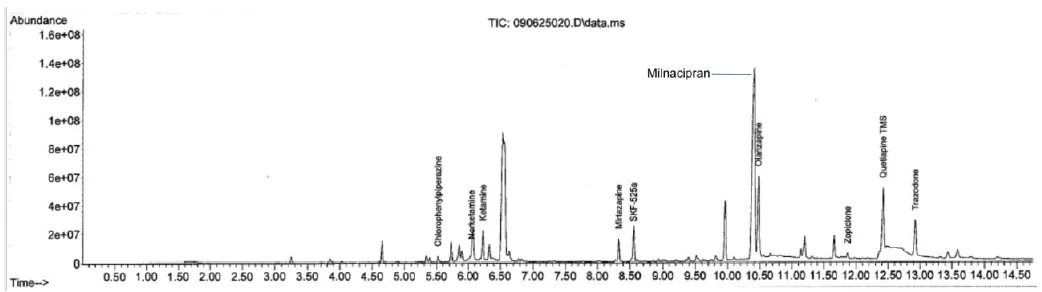
Chromatogram demonstrating a peak consistent with a low concentration of propranolol. Amlodipine and venlafaxine were not detected. (The study was unable to distinguish dextro and levo isomers of milnacipran, zopiclone); neither could specific serum concentrations be obtained.)

**Table:** Venoarterial extracorporeal membrane oxygenation parameters.

Patient Factors	
Height (cm)	160
Weight (kg)	73.9
Extracorporeal Membrane Oxygenation Configuration	
Configuration	Single-lumen cannulae
Cannulation site	Right femorofemoral
Drainage tip position	Inferior cavoatrial junction
Cannula dimensions	V25/55 - A19/15
RPM / Flow	3000 / 3.75 L/min
Sweep gas	1 L/min

Cannula dimensions reflect outer diameter in French sizing and cannula length in centimeters.

*A*, arterial; *RPM*, rotations per minute; *V*, venous.
